# New York State Veterinary Medical Society

**Published:** 1891-07

**Authors:** 


### NEW YORK STATE VETERINARY MEDICAL SOCIETY.

It has been decided by the officers of the New York State Veterinary Medical Society to postpone the holding of the semi-annual meeting of the society until Wednesday morning, August 12, 1891, at nine o'clock. It has also been decided to hold the meeting in New York city to accommodate the members from the eastern part of the state and to comply with the requests of several veterinarians of New York and Brooklyn who are taking considerable interest in matters pertaining to Veterinary Legislation, an article on which was published in the June number of this JOURNAL.

